# Supplementary material for: Binding Networks Identify Targetable Protein Pockets for Mechanism-Based Drug Design
Source: Int J Mol Sci. 2022 Jun 30;23(13):7313. doi: 10.3390/ijms23137313 (PMC9267029; doi:10.3390/ijms23137313)
Supplement: Supplementary file 1 [file ijms-23-07313-s001.zip › blebbi_ijms_supporting.pdf]

Supplementary Information for

Binding networks identify targetable protein pockets for mechanism-based drug design

Mónika Bálint[1#], Balázs Zoltán Zsidó[1#], David van der Spoel[2], Csaba Hetényi[1,*]

[1]*Pharmacoinformatics Unit, Department of Pharmacology and Pharmacotherapy, Medical School, University of Pécs, Szigeti út 12., 7624 Pécs, Hungary.*
[2]*Department of Cell and Molecular Biology, Uppsala University, Box 596, SE-75124 Uppsala, Sweden.*

*Corresponding author.
E-mail: hetenyi.csaba@pte.hu
#Equal contribution.

This PDF file includes:

Supplementary text (Results)

Figures S1 to S3

Tables S1 to S8

Legends for Movies S1 to S2

SI References

Other supplementary materials for this manuscript include the following:

Movies S1 to S2

Supplementary Results

1 Re-docking of inhibitors

Re-docking of ligands BS and PBP were performed to the holo myosin 2 structures (PDB IDs 1yv3 and 2jhr) used as targets. During target preparation, water molecules, co-crystalized ions and ligand molecules were removed from the PDB structures. The target structures were then subjected to energy minimization as they were for the apo structures, with the difference that the heavy atoms of the myosin 2 structure were restrained. After target minimization, target molecules were prepared and docking was performed as described in the main text Methods Section "Prerequisite binding modes" with the following modifications on the protocol. The grid parameters were changed to a box of 80x80x80 grid points with 0.375 Å spacing. The center of the grid box was set to that of the destination BS conformation (PDB ID 1vy3) and the experimental PBP conformation (PDB ID 2jhr). Ten docking runs were performed. The conformations of PBS and BS were evaluated with the best binding energy obtained after the focused docking on the targets. Root mean squared deviations (RMSD) of the atomic coordinates were calculated to quantify the differences between the calculated docked conformations (C) and the experimental reference ligand conformations (R) according to Eq. 1.

$$\mathbf{RMSD} = \sqrt{\frac{\mathbf{1}}{\mathbf{NH_L}}\sum_{\mathbf{i=1}}^{\mathbf{NH_L}}|\mathbf{C_i} - \mathbf{R_i}|^{\mathbf{2}}} \qquad \text{Eq. (1)}$$

where NH_L is the number of ligand heavy atoms, R is the space vector of the ith heavy atom of the reference ligand molecules, and C is the space vector of the ith heavy atom of the calculated ligand conformation. The RMSD values of **Table S1** show good agreement between experimental and docked ligand binding modes and the results are also presented in **Figure S1** for BS.

Table S1 RMSD values measured between re-docked and experimental inhibitor conformations

**Inhibitor**	**RMSD (Å)**	**Reference PDB structure with inhibitor bound to myosin 2**
BS	0.9	1yv3
PBP	2.8	2jhr

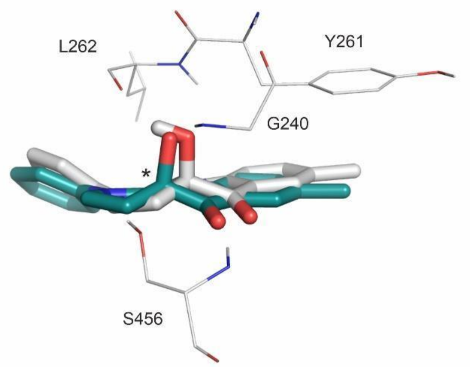

RMSD (Å)	0.9
E_{inter} (kcal/mol)	-46.1
IC_{50} (μM)	2.2

Figure S1 The figure shows the destination binding mode (D) of BS produced by re-docking to the holo conformation of the non-muscular myosin 2 (PDB code 1yv3) (thick stick figure in grey). Amino acids of the myosin 2 binding pocket are shown as thin grey sticks. The crystallographic destination binding mode of BS (PDB code 1yv3, teal sticks) is also shown for comparison to the docked BS conformation.

2 Wrapping

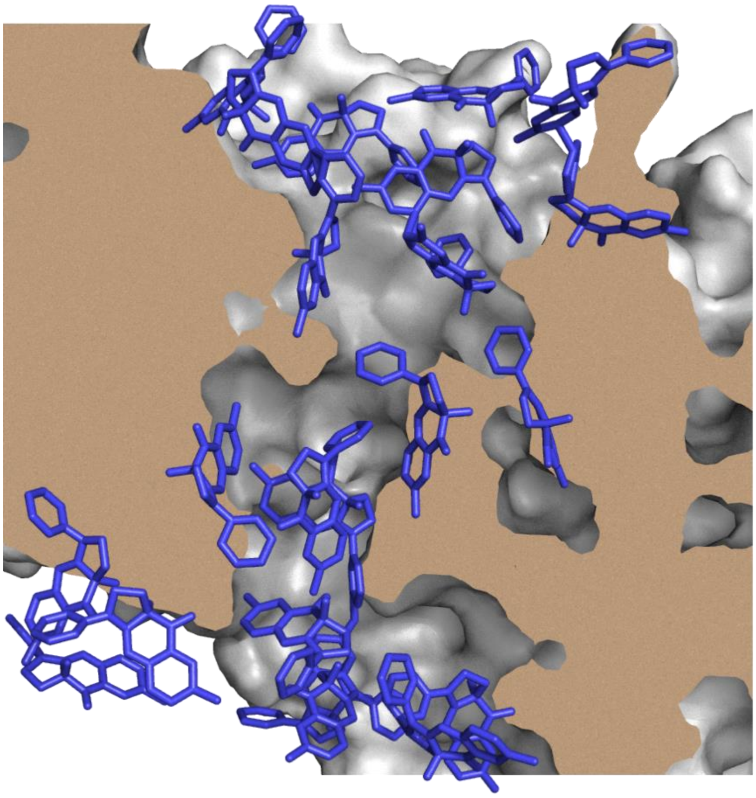

Figure S2 The monolayer of 16 docked conformations (blue sticks) wrapping the entire inner surface of the myosin 2 binding cavity. The cross-section of the cavity is shown in grey surface. Files of the wrapping step are provided as on-line Supplementary Information at https://zenodo.org/record/6536287#.YnzSsFRByHt

3 Generation of PMs: MD simulations

Table S2 Closest d_D values obtained after 16 parallel MD simulations performed on myosin 2-BS complexes obtained from the wrapping step used as starting structures of the MD simulations. Sample files of the MD simulations are provided as on-line Supplementary Information at https://zenodo.org/record/6536287#.YnzSsFRByHt

**MD simulation #**	**Time (ns) until d_D > 30 Å**	**d_D (Å)**
1	1000	4.8
2	1000	13.3
3	1000	9.5
4	639	26.4
5	11	29.9
6	7	22.4
7	1000	10.5
8	127	28.0
9	1	28.4
10	1000	6.4
11	4	26.1
12	62	29.1
13	1000	8.4
14	9	22.4
15	26	25.0
16	43	25.0

Note. MD simulations were carried out for a maximum of 1000 ns, if BS did not leave the binding cavity (d_D remained < 30 Å) of myosin 2.

4 Generation of PMs: Clustering results

Table S3 The interaction energy (E_{inter}) and d_D values obtained for the PMs after clustering of the conformation pool. Files of the pool and clustering are provided as on-line Supplementary Information at https://zenodo.org/record/6536287#.YnzSsFRByHt

PM #	E_{inter} (kcal/mol)	d_D (Å)
1	-50.09	6.9
2	-45.85	8.9
3	-40.74	6.0
4	-33.34	15.7
5	-32.50	13.6
6	-29.60	9.1
7	-28.02	13.5
8	-27.81	17.1
9	-25.68	17.0
10	-23.21	14.4
11	-22.01	23.3
12	-21.66	24.4
13	-20.99	15.0
14	-19.26	18.1
15	-18.85	26.3
16	-16.75	18.6
17	-16.60	29.2
18	-12.94	15.2
19	-11.77	28.4
20	-8.99	25.5
21	-8.94	24.3
22	-7.13	22.3
23	-0.28	29.9

5 Binding graph

Table S4 Pairwise connectivity list of the binding graph calculated from the coordinates of PMs. Edges of the binding graph was drawn according to the distances between the PM pairs (d_{PM}). For information, d_D values are also listed for both PMs.

**PM #**	**PM #**	**d_D (Å)**	**d_D (Å)**	**d_{PM} (Å)**
0(D)	3	0.00	5.94	5.94
0(D)	1	0.00	6.91	6.91
0(D)	2	0.00	8.91	8.88
0(D)	6	0.00	9.10	9.08
1	3	6.91	5.94	9.39
1	5	6.91	13.61	10.26
2	6	8.91	9.10	4.10
2	3	8.91	5.94	8.85
2	4	8.91	15.72	9.79
2	10	8.91	14.42	9.80
2	18	8.91	15.22	11.15
2	8	8.91	17.11	11.83
3	6	5.94	9.10	6.95
3	5	5.94	13.61	10.51
3	9	5.94	16.94	11.37
4	6	15.72	9.10	6.84
4	10	15.72	14.42	9.61
4	22	15.72	22.34	10.08
4	12	15.72	24.34	10.54
5	14	13.61	18.06	4.78
5	9	13.61	16.94	9.05
5	16	13.61	18.58	9.66
5	11	13.61	23.32	10.79
6	10	9.10	14.42	8.76
7	18	13.50	15.22	6.67
7	13	13.50	15.01	7.19
7	8	13.50	17.11	10.30
8	18	17.11	15.22	5.48
9	20	16.94	25.54	9.14
9	14	16.94	18.06	9.38
9	11	16.94	23.32	11.48
10	18	14.42	15.22	9.19
10	22	14.42	22.34	9.24
10	13	14.42	15.01	9.81
11	17	23.32	29.14	7.21
11	20	23.32	25.54	8.45
11	14	23.32	18.06	8.89
11	19	23.32	28.40	11.52
12	22	24.34	22.34	6.29
12	15	24.34	26.26	9.27
13	18	15.01	15.22	9.56

14	16	18.06	18.58	9.05
14	21	18.06	24.27	10.83
14	20	18.06	25.54	11.79
15	22	26.26	22.34	11.57
16	21	18.58	24.27	9.63
17	20	29.14	25.54	7.97

6 Final docking

Table S5 Final d_D values obtained after docking of BS by 1-µs-long simulated annealing MD simulations. All MD simulations were started from PM_1 started with different initial velocity distributions. Sample files of the MD simulations are provided as on-line Supplementary Information at https://zenodo.org/record/6536287#.YnzSsFRByHt

MD #	d_D (Å)
1	0.9
2	5.3
3	51.1
4	28.3
5	3.9
6	3.1
7	16.9
8	27.2
9	6.9
10	1.5
11	4.1
12	7.8

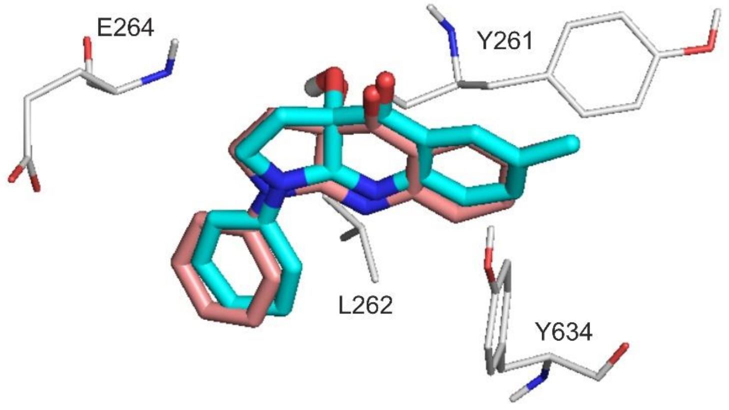

Figure S3 Structure of the BS binding mode after Final Docking (FD_6) (teal sticks), and the experimental binding position of BS (salmon sticks, from PDB 1yv3. Interacting amino acids of the myosin 2 protein are shown as grey sticks and labelled accordingly.

Supplementary Methods

Table S6 Amino acids constituting the binding cavity of myosin 2 (Numbering follows that of PDB structure 1mmd.)

SER	181
LYS	185
THR	230
THR	231
ARG	232
ASN	233
SER	237
ARG	238
PHE	239
GLY	240
LYS	241
PHE	242
SER	257
ILE	258
GLN	259
SER	260
TYR	261
LEU	262
LEU	263
GLU	264
LYS	265
SER	266
ARG	267
VAL	269
PHE	270
GLN	271
SER	272
GLU	275
SER	416
ALA	420
LYS	423
ALA	424
GLY	427
ARG	428
PHE	430
LEU	431
VAL	434
LYS	435
ASN	438
ASP	454
ILE	455
SER	456
GLY	457

GLU	459
PHE	461
LYS	462
VAL	463
ASN	464
SER	465
PHE	466
GLU	467
GLN	468
ILE	471
GLU	580
ILE	581
GLN	582
ASP	583
GLU	586
LYS	587
ASP	590
PRO	591
PRO	615
ASN	616
ILE	617
SER	619
ARG	620
ALA	621
NME	622
VAL	630
GLN	633
TYR	634
GLN	637

Table S7

Atoms for which distance restraint was applied

Mg^{2+}	OA1 (Pi)
Mg^{2+}	O2B (ADP)
Mg^{2+}	PB (ADP)
Mg^{2+}	PA (Pi)
OA1 (Pi)	O2B (ADP)
OA1 (Pi)	PB (ADP)
OA1 (Pi)	PA (Pi)
O2B (ADP)	PB (ADP)
PB (ADP)	PA (Pi)

Table S8

Temperature scheme applied for the simulated annealing simulations

Annealing time (ps)	0	10000	25000	50000	60000	500000	940000	950000	975000	990000	1000000
Annealing temp (K)	300	315.15	330.15	343.15	353.15	353.15	353.15	343.15	330.15	315.15	300.00

Legends for Movies

Movie S1. The suggested binding pathway of BS to myosin 2. The protein is shown as grey cartoon. ADP is shown as grey all atom representation sticks. The experimental binding mode of BS (D) is shown as salmon all atom representation sticks, and the PMs are shown as teal all atom representation sticks. The orange arrow indicates the movement of BS from the parking lot towards the destination binding site, and teal spheres indicates PMs along the way.

Movie S2. Final docking (FD) stages of BS. The flipping of BS and the consequential movement of L262, that allows BS into the final, destination binding site. Note, the different nomenclature of the binding modes. FD starts from PM_1 (PM_1=FD_1) and finishes with D (D=FD_6). Myosin 2 is shown as grey cartoon. ADP is shown as grey all atom representation sticks. The experimental binding mode of BS (D) is shown as salmon all atom representation sticks, and the FDs are shown as teal all atom representation sticks. The red arrow indicates the movement of the L262 amino acid.

References

[1] Verhasselt S, Roman BI, De Wever O, Van Hecke K, Van Deun R, Bracke ME, et al. Discovery of (S)-3′-hydroxyblebbistatin and (S)-3′-aminoblebbistatin: polar myosin II inhibitors with superior research tool properties. Org Biomol Chem 2017;15:2104–18. doi:10.1039/c7ob00006e.

[2] Fedorov R, Böhl M, Tsiavaliaris G, Hartmann FK, Taft MH, Baruch P, et al. The mechanism of pentabromopseudilin inhibition of myosin motor activity. Nat Struct Mol Biol 2009;16:80–8. doi:10.1038/nsmb.1542.

[3] Allingham JS, Smith R, Rayment I. The structural basis of blebbistatin inhibition and specificity for myosin II. Nat Struct Mol Biol 2005;12:378–9. doi:10.1038/nsmb908.
